# Effect of Cooperation Level of Group on Punishment for Non-Cooperators: A Functional Magnetic Resonance Imaging Study

**DOI:** 10.1371/journal.pone.0041338

**Published:** 2012-07-23

**Authors:** Fumitoshi Kodaka, Hidehiko Takahashi, Makiko Yamada, Harumasa Takano, Kazuhiko Nakayama, Hiroshi Ito, Tetsuya Suhara

**Affiliations:** 1 Clinical Neuroimaging Team, Molecular Neuroimaging Program, Molecular Imaging Center, National Institute of Radiological Sciences, Inage-ku, Chiba, Japan; 2 Department of Psychiatry, Jikei University School of Medicine, Minato-ku, Tokyo, Japan; 3 Department of Psychiatry, Kyoto University Graduate School of Medicine, Sakyo-ku, Kyoto, Japan; 4 Precursory Research for Embryonic Science and Technology, Japan Science and Technology Agency, Saitama, Japan; 5 Biophysics Team, Molecular Neuroimaging Program, Molecular Imaging Center, National Institute of Radiological Sciences, Inage-ku, Chiba, Japan; Chiba University Center for Forensic Mental Health, Japan

## Abstract

Sometimes we punish non-cooperators in our society. Such behavior could be derived from aversive emotion for inequity (inequity aversion) to make non-cooperators cooperative. Thus, punishing behavior derived from inequity is believed to be important for maintaining our society. Meanwhile, our daily experiences suggest that the degree of cooperation by the members of society (cooperation level of the group) could change the punishing behavior for non-cooperators even if the inequity were equal. Such effect of the cooperation level of the group cannot be explained by simple inequity aversion. Although punishment-related brain regions have been reported in previous functional magnetic resonance imaging (fMRI) study, little is known about such regions affected by the cooperation level of the group. In the present fMRI study, we investigated the effect of the cooperation level of the group on the punishing behavior for non-cooperators and its related brain activations by a paradigm in which the degree of the cooperative state varied from low to high. Punishment-related activations were observed in brain regions such as the anterior insula, dorsolateral prefrontal cortex (DLPFC), and anterior cingulate cortex (ACC). The quantity of punishment in a high cooperation context was greater than in a low cooperation context, and activation in the right DLPFC and ACC in a high cooperation context showed greater activity than in a low cooperation context. This indicates that the cooperation level of the group, as well as aversive emotion for inequity, is the important factor of punishing behavior.

## Introduction

Sometimes we punish non-cooperators in our society. Such punishing behavior could be derived from aversive emotion for inequity [1], [2], [3], [4]. Previous studies suggest that members of society were willing to punish non-cooperators even if punishment entails costs for the punishers to make non-cooperators cooperative, indicating that punishment derived from such inequity aversion could play an important role in the maintenance of human society [5].

On the other hand, our daily experiences indicate that the degree of cooperation by the members of society (cooperation level of the group) could modulate the punisher’s emotions towards non-cooperators. For instance, if an observer must punish a passerby who ignored a red signal, aversive emotions towards him or her would be severe in a situation in which the rest of the passersby wait before the signal, more than in a situation in which they also choose to ignore it. Critical mass, a point at which the number of non-cooperators suddenly surges while their number is gradually increasing [6], partly explains the phenomenon of a low cooperative level decreasing punishing emotions towards non-cooperators. In such a context, severe punishment tends to be given to a non-cooperator if the rest of group members behave cooperatively, whereas weaker punishment tends to be given to them if they also disregard cooperation.

The cooperation level of the group may thus be an important modulator of punishment, and the neural correlates of punishment could be modulated by the context as well. The neural basis of punishment derived from inequity aversion has been studied using functional magnetic resonance imaging (fMRI) through such as the Ultimatum Game [3], [7]. In that study, activations of the anterior insula (AI), dorsolateral prefrontal cortex (DLPFC), and anterior cingulate cortex (ACC) were observed. However, little is known about which of the punishment-related brain regions (AI, DLPFC, ACC) are modulated by the cooperation level.

The purpose of the study was to investigate the effect of the cooperation level of the group on punishing behavior for non-cooperators, and the effect on brain activation of the punishment-related brain regions.

## Methods

### Ethics Statement

In accordance with the Helsinki Declaration of Human Rights (2000), written informed consent was obtained from all volunteers after detailed explanation of the study. This study protocol was approved by the Ethics and Radiation Safety Committees of the National Institute of Radiological Sciences, Chiba, Japan.

### Subjects

Nineteen healthy men originally participated in the study. However, two were excluded from the analysis due to corrupted image files for one, and misunderstanding of the experimental task for the other. Consequently, seventeen men [mean age 26.4±(SD) 5.0 years] continued on to the image analytic procedure. All of the subjects were right-handed.

The reason for the single gender was that gender could have an effect on aversive emotion for inequity [8], [9] or on the strategy for playing economic games [10]. They did not have any history of psychiatric or neurological disorders, substance abuse, or physical disease.

### Materials

**Figure 1:**
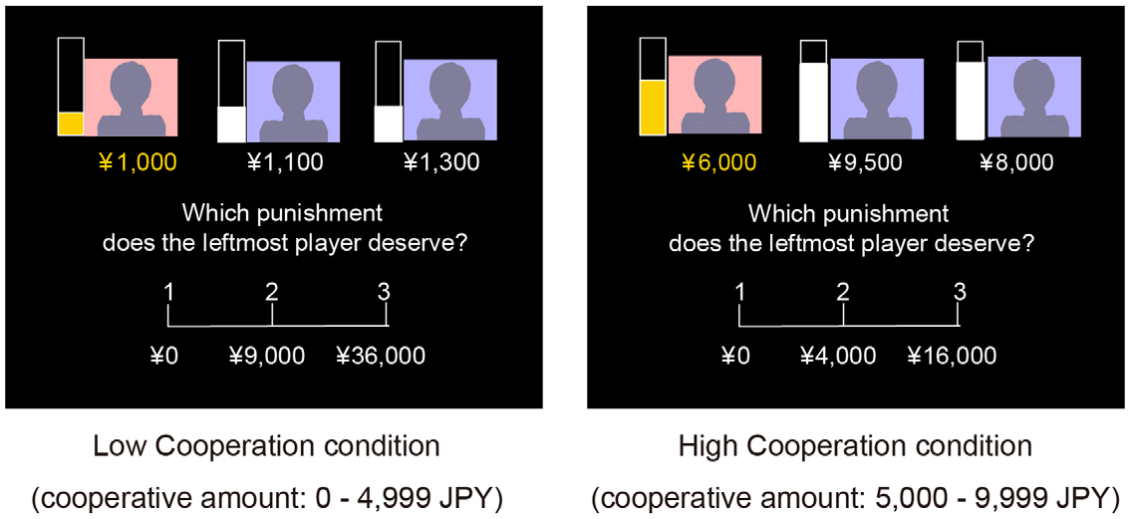
Details of the behavioral paradigm. Each cooperation condition was defined according to the average amount of the personas’ contributions other than the specific non-cooperator. Subjects were asked to punish the leftmost persona with three alternatives. Low Cooperation level condition (Left) and High Cooperation level condition (Right) are presented.

Each subject was presented with a scene on a screen in which three personas contribute money (Figure 1). These three personas simultaneously each contribute a certain amount of money up to a ceiling of 10,000 Japanese yen (JPY) (about $120). Among the three personas (for descriptive purposes, A, B, C from left on the screen), “A” always made the lowest contributions (the non-cooperator), whereas both “B” and “C” always made similar contributions, with the actual amounts depending on the experimental conditions. Their contributions defined the cooperation level of the group.

#### Definition of cooperation level of the group

The cooperation level of the group was defined as an averaged amount of the contributions of personas “B” and “C”. Two conditions of the cooperation level were prepared, i.e., HC (high cooperation level condition) 5,000–9,999 JPY and LC (low cooperation level condition), 0–4,999 JPY. We also used CC (control cooperation level condition), in which all 3 personas contributed 10,000 JPY each. The ranges of contributions of the non-cooperator in HC, LC, and CC were 5000–6000 JPY, 800–1500 JPY, and 0 JPY. Although another HC condition, in which the contribution of “A” was almost zero (range: 100–1000 JPY), and contributions of the cooperators were the same range as those of HC was prepared to confirm the effect of inequity on punishment-related brain regions, this condition was not analyzed in the present study because sufficient brain activation was observed in HC and LC.

**Table 1:** Brain activations in High Cooperation condition and Low Cooperation condition relative to Control Cooperative condition.

Brain region	Coordinates			BA	Z-score
	*x*	*y*	*Z*		
*High Cooperation minus Control*					
L DLPFC	−48	9	27	9	4.99
	−44	24	23	46	4.33
R DLPFC	50	11	25	9	4.2
L OFC	−40	41	2	10	4.91
L Insula	−32	17	−4	47	4.16
	−32	17	−14	47	3.83
R Insula	34	23	−10	47	4.29
	32	23	−3	13	4.19
	44	16	3	13	4.15
L ACC	−4	21	41	32	5.27
	−10	25	28	32	4.57
R ACC	8	25	28	32	4.38
L IPL	−34	−58	42	7	6.41
	−44	−41	41	40	6.31
R IPL	42	−47	39	40	4.88
	53	−36	50	40	4.89
L Precuneus	−26	−70	29	19	6.23
R Precuneus	28	−70	37	19	6.38
	4	−73	48	7	4.99
R Cuneus	8	−76	35	19	4.82
L Postcentral gyrus	−40	−24	60	3	5.24
*Low Cooperation minus Control*					
L DLPFC	−46	9	29	9	5.48
	−44	23	23	46	4.4
R DLPFC	46	32	28	9	4.37
	46	9	22	9	3.75
	40	43	7	46	3.63
L OFC	−42	41	0	10	5.01
L Insula	−32	21	−8	47	4.03
R Insula	34	23	1	13	4.28
L ACC	−8	25	37	32	4.65
R ACC	2	20	45	8	4.04
L IPL	−46	−41	43	40	4.86
L Precuneus	−32	−60	40	19	4.81

L, left; R, right; AI, anterior insula; DLPFC, dorsolateral prefrontal cortex; ACC, anterior cingulate cortex; OFC, orbitofrontal cortex; IPL, inferior parietal lobule. Coordinates and Z-score refer to the peak of each brain region.

#### Punishment for non-cooperator

The subjects were asked to assume a background story that the three personas contributed money to build a public facility, and that they knew each other’s contributions. One experimenter explained that the players actually did not exist, and thus deception was not performed. They were asked to punish the non-cooperator by choosing from three alternatives as penalty, i.e., 1. punish no one (No Punishment), 2. punish the amount the non-cooperator did not contribute (Moderate Punishment), and 3. punish four-fold of the second alternative (Severe Punishment) within 6000 ms. The obtained behavioral data were transformed to parametric variables, i.e., No Punishment = 0, Moderate Punishment = 1, and Severe Punishment = 2, and submitted to the analytic procedure.

**Figure 2:**
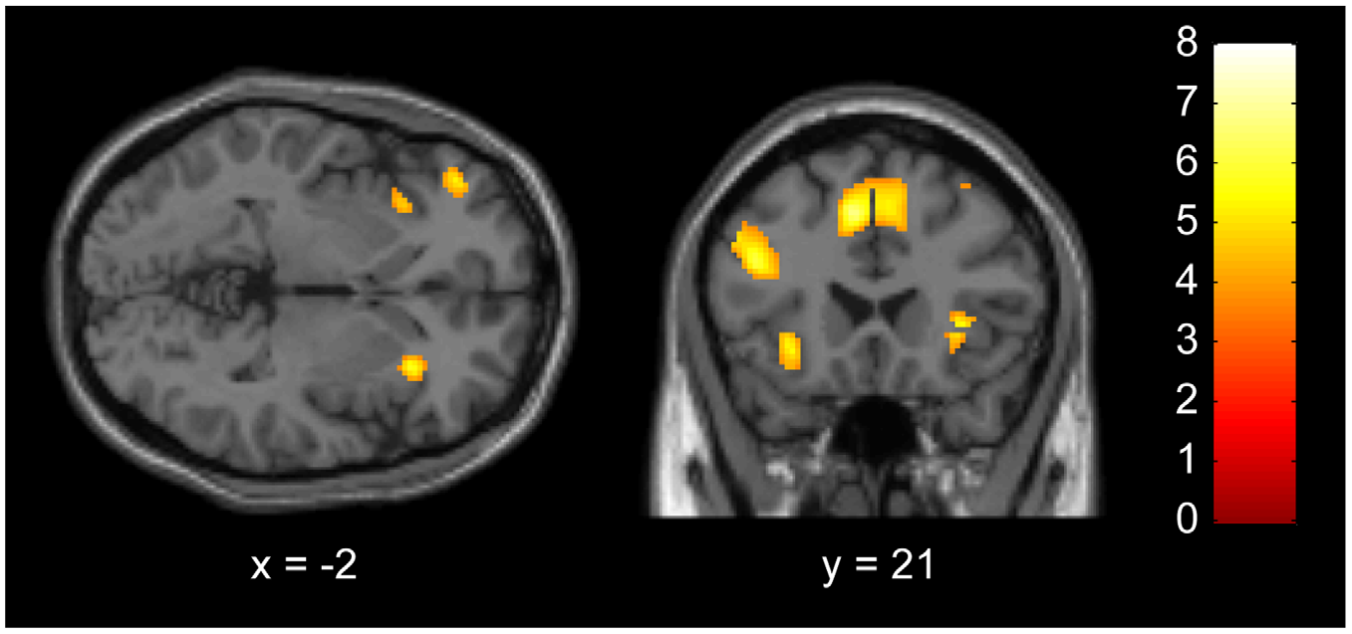
The common brain activations in High minus Control Cooperation and Low minus Control Cooperation contrasts. Significant activations were observed in the bilateral AI, bilateral DLPFC, and OFC (P<0.001, uncorrected for display use). AI, anterior insula; DLPFC, dorsolateral prefrontal cortex; OFC, orbitofrontal cortex.

**Table 2:** Common brain activations in High minus Control Cooperation contrasts and Low minus Control Cooperation contrasts.

Brain region	Coordinates			BA	Z-score
	*x*	*y*	*Z*		
*High Cooperation minus Control and Low Cooperation minus Control*					
L DLPFC	−48	9	27	9	4.99
	−44	23	23	46	4.28
R DLPFC	46	35	30	9	4.01
	46	9	22	9	3.75
L OFC	−42	41	2	10	4.74
L Insula	−30	21	−8	47	3.97
R Insula	32	23	1	13	4.1
L ACC	−8	23	39	32	4.54
R ACC	2	20	45	8	4.04
L IPL	−46	−41	43	40	4.86
L Precuneus	−32	−60	40	39	4.81

L, left; R, right; AI, anterior insula; DLPFC, dorsolateral prefrontal cortex; ACC, anterior cingulate cortex; OFC, orbitofrontal cortex; IPL, inferior parietal lobule. Coordinates and Z-score refer to the peak of each brain region.

### Image Acquisition

MR images were acquired with a 3.0-T Signa system (General Electric, Milwaukee, WI) equipped with an 8-channel phased array head coil. Functional images of 518 volumes were acquired with T2*-weighted gradient echo planer imaging sequences sensitive to BOLD contrast. Each volume consisted of 28 transaxial contiguous slices with a slice thickness of 4.2 mm to cover almost the whole brain (flip angle, 90°; TE, 30 ms; TR, 2000 ms; matrix, 64×64; field of view, 24×24 cm).

**Figure 3:**
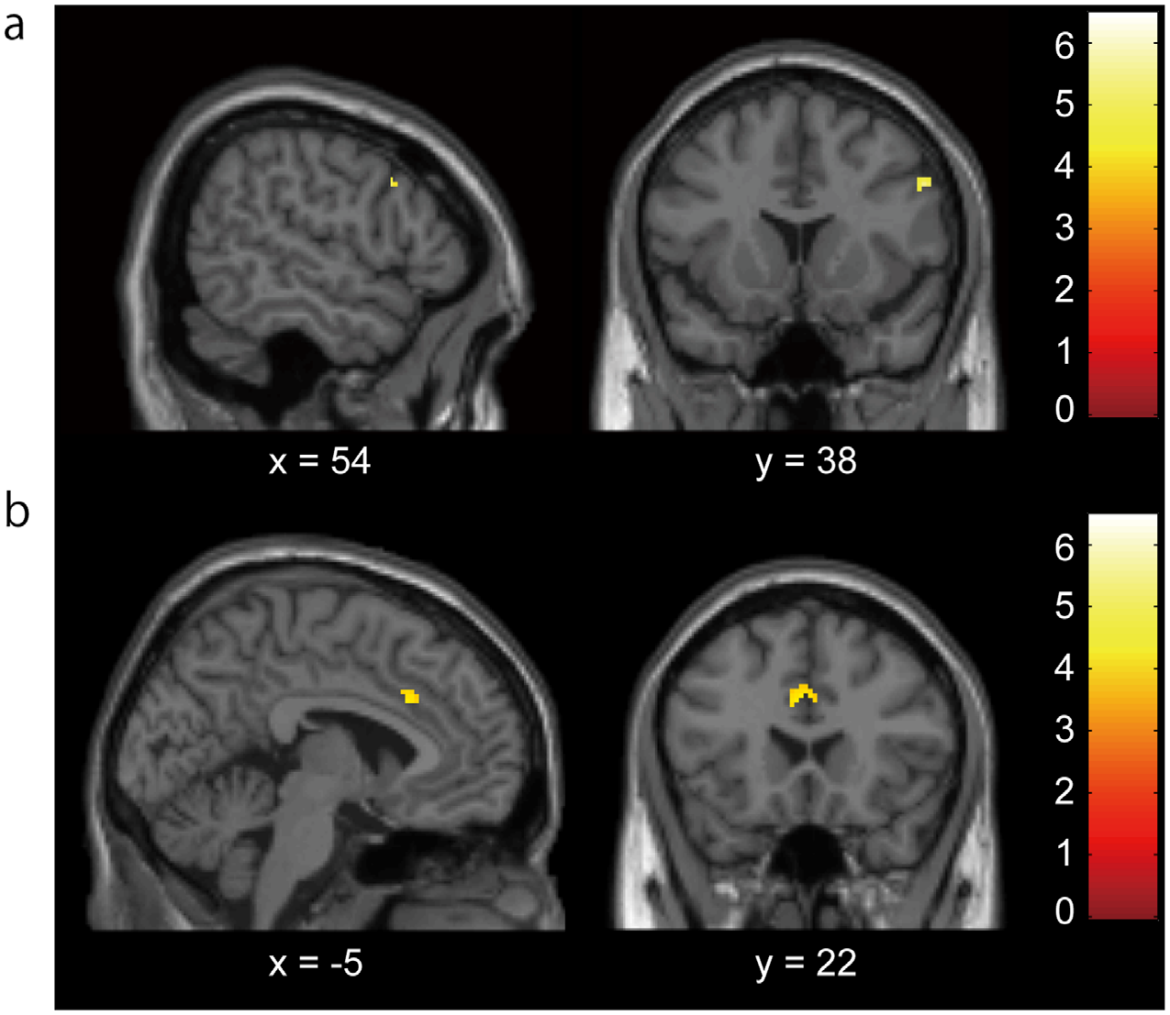
The brain activation in High Cooperation minus Low Cooperation contrast. Significant activations observed in the right DLPFC (P<0.001, uncorrected for display use) (a) and left ACC (b). ACC, anterior cingulate cortex, DLPFC, dorsolateral prefrontal cortex.

**Table 3:** Brain activations in High minus Low Cooperation contrasts.

Brain region	Coordinates			BA	Z-score
	*x*	*y*	*Z*		
*High Cooperation minus Low Cooperation*					
R DLPFC	55	11	27	9	4.72
L ACC	−4	23	27	32	3.34

L, left; R, right; DLPFC, dorsolateral prefrontal cortex; ACC, anterior cingulate cortex; MFG, middle frontal gyrus; IFG, inferior frontal gyrus. Coordinates and Z-score refer to the peak of each brain region.

Visual stimuli were projected onto a screen mounted on a head coil via a projector installed in the MRI room. Each subject was asked to watch the scene of contribution, and to determine the degree of punishment by pressing buttons. One session comprised 4 conditions (HC, LC, CC, and unanalyzed condition (UC)), and for each condition 5 punishing events in which each persona’s contributions were set randomly within the predetermined cooperation level were used 5 times, yielding 100 events in total. All the stimuli were presented in an event-related paradigm, with each event in pseudo-random order for 6000 ms. The time duration for consideration of the quantity of punishment was fixed for 6000 ms in each event. The inter-stimulus interval (ISI) was 2000–4000 ms. During the ISI period, the subjects viewed a crosshair pattern projected to the center of the screen. Immediately after the subjects made choices, the rest of the screen display replaced to the crosshair screen in order to minimize decision-making biases.

### Analysis of Functional Imaging Data

#### Image pre-processing

Data analysis was performed with the statistical parametric mapping software package SPM5 (Wellcome Department of Cognitive Neurology, London, UK) running on MATLAB (Mathworks, MA, USA).

All volumes were corrected for sequential slice timing, and were realigned to the first volume of the session to correct for motion effects. The realigned images were spatially normalized to standard space as defined by the Montreal Neurological Institute (MNI) template. After normalization, all scans had a resolution of 2×2×2 mm^3^. Subsequently, all normalized images were spatially smoothed with a 3D isotropic Gaussian kernel (full-width half-maximum of 8 mm). Low-frequency noise was removed by applying a high-pass filter (cutoff period  = 128 s) to the fMRI time scale at each voxel. A temporal smoothing function was applied to the fMRI time series to enhance the temporal signal-to-noise ratio.

#### First-level analysis

Event-related hemodynamic changes on punishment for each condition were examined using general linear with boxcar functions convolved with a hemodynamic response function. Statistical parametric maps for each contrast of the t-statistics were calculated on a voxel-by-voxel basis. We contrasted HC with CC and LC with CC to examine neural activation by punishment in AI, DLPFC, and ACC. Then, to examine the effect of the cooperative level on the punishment-related brain regions, we contrasted HC with LC and LC with HC.

#### Second-level analysis

A random effects model, which estimates the error variance for each condition across the subjects, was implemented for group analysis. This procedure provides better generalization for the population from which data are obtained. The contrast images from single-subject analysis were entered into group analysis.

A one-sample t test was applied to determine group activation for each effect. A statistical threshold of *p*<0.05 corrected for multiple comparisons across the whole brain was used, except for an *a priori* region-of-interest that included the punishment-related brain regions AI, DLPFC, and ACC. These regions were chosen according to a previous study on neural correlates of inequity aversion [3]. We applied SPM’s small volume correction to correct for multiple testing in regions about which we had an *a priori* hypothesis. These *a priori* regions were defined by standard templates implemented in brain atlas software [11]. We described activations surviving a threshold of *p*<0.05 with an extent threshold of 5 contiguous voxels.

To examine common punishment-related brain regions in HC and LC, conjunction analysis between the HC-CC and LC-CC contrasts was performed. Then, to examine the effect of the cooperation level on punishment-related brain regions, the HC-LC and LC-HC contrasts were examined. The HC-CC contrast (*p*<0.001, uncorrected) was used as a mask, overlaid on the HC-LC contrast. Similarly, the LC-CC contrast (*p*<0.001, uncorrected) was also used as a mask, overlaid on the LC-HC contrast. The HC-CC and LC-CC contrasts were used to limit the punishment-related brain regions.

## Results

### Behavioral Results

A significant effect of the cooperation level of the group on punishment was observed. The mean amounts of actual amount of punishment in HC, LC, and CC were 4740 JPY (SD = 1793) in HC, 1772 JPY (SD = 2830) in LC, and 0 JPY (SD = 0) in CC, respectively. The converted mean amounts of each punishment in HC, LC, and CC in the seventeen subjects were 0.97 (SD = 0.21), 0.18 (SD = 0.30), 0 (SD = 0) respectively.

The differences in converted mean punishment values between HC and LC conditions were statistically significant by paired *t*-test (*t* = 9.97; *p*<0.05).

### fMRI Results

#### Brain activations by punishing behavior

Punishing behavior produced activations in various brain regions related to punishment for HC-CC and LC-CC contrasts. The HC-CC contrast produced activations in the bilateral DLPFC, bilateral AI, bilateral ACC, orbitofrontal cortex (OFC), bilateral inferior parietal lobule (IPL), bilateral precuneus, right cuneus, and left postcentral gyrus. The LC-CC contrast produced activations in the bilateral DLPFC, bilateral AI, bilateral ACC, left OFC, left IPL, and left precuneus (Table 1).

HC-CC and LC-CC contrasts also had common activation in the reported punishment-related regions. Conjunction analysis of the HC-CC and LC-CC contrasts produced activations in the bilateral DLPFC, bilateral AI, bilateral ACC, left OFC, left IPL, and left precuneus (Figure 2, Table 2).

#### Effect of cooperation level of the group on the punishment-related brain regions

The HC level had greater effect on DLPFC and ACC. The HC-LC contrast with HC-CC mask produced activations in the right DLPFC and left ACC (Figure 3, Table 3). We found overwrapped activations between the regions in the conjunction analysis and those in HC-LC contrast. We had 176 mm^3^ (22 voxel) of overwrapped region in the right DLPFC and 400 mm^3^ (50 voxel) in the left ACC (*p*<0.001, uncorrected for conjunction analysis; *p*<0.005, uncorrected for HC-LC contrast).

On the other hand, we found no effect of the LC level on punishment-related brain regions. The LC-HC contrast with LC-CC mask did not produce significant brain activations.

## Discussion

We found a significant effect of the cooperation level of the group on punishing behavior for non-cooperators, and that the effect on neural activation was significant in the right DLPFC and left ACC.

### Relation between Behavioral Measurements and Regional Activations

The behavioral results showed that the punishment in HC condition was greater than in LC condition, indicating that the cooperation level of the group affects the degree of the punishment. Accordingly, the punishment-related brain regions in both HC and LC conditions showed different activations. The present study thus demonstrated the effect of the cooperation level of the group on the punishment related-brain regions. Conjunction analysis between the HC-CC and LC-CC contrasts produced activation in the bilateral DLPFC, bilateral AI, left OFC, and bilateral ACC. These findings are in agreement with previous studies on punishment derived from inequity [3], [7], [12], [13].

### Effect of Cooperation Level of the Group on Punishment-related Brain Regions

In HC condition, greater right DLPFC and left ACC activation was induced in response to non-cooperator than in LC condition, whereas no active brain regions were observed in LC-HC contrast. In this analysis, the difference could be referred to as the component of the punishment modulated by the cooperative social context. In other words, this component may be regarded as context-dependent punishment, and it is different from the punishment derived from inequity. Although the DLPFC has been linked to various cognitive processes such as executive control [14], [15], the decision-making process in a social context also belongs to one of such processes [16]. In the present study, the cooperation level of the group may work as a social contextual stimulus, leading to a change of the BOLD signal in the right DLPFC, i.e., the right DLPFC was recruited to punish the non-cooperator on the basis of the cooperative social context. Although there is little evidence to explain the effect of the cooperative social context on brain function, lesions and neuroimaging studies on moral cognition [17] may help us discuss the effect of the cooperative social context. Studies in patients with prefrontal lesions at early developmental stage [18], [19] and studies of patients with frontotemporal dementia [20], [21] reported that functional deficit in the right DLPFC leads to disturbance of moral cognition. These studies may support our result that the right DLPFC is modulated by the cooperative social context.

Furthermore, it is reported that the right DLPFC is recruited when subjects punish protagonists in a scenario with criminal responsibility compared to one without [22]. Punishments based on criminal responsibility should take the social context into account. For instance, driving under the influence was not uncommon a few decades ago, and such violation was not necessarily harshly condemned. However, this situation has changed, and the majority of society members (in Japan and U.S.) conform to the rules. The individual driving under the influence is strictly condemned these days. The DLPFC recruitment in our study suggests the importance of the social context in third-party punishment based on the individual’s responsibility.

### Limitations

The current study has some limitations. Although we carefully controlled the cooperation level of the group by the contributions of the cooperators, the inequity between the non-cooperator and the cooperators could not be equal among experimental conditions. This could be a confounding factor in investigation of the effect of the cooperation level of the group on punishment-related brain regions. This also indicates that we could not discuss the relationship between the degree of inequity and observed brain activations in all the experimental conditions. The previous study reports that degree of inequity could be related only with AI [3]. Therefore the right DLPFC and the ACC activation observed in this study might not be affected by the degree of inequity, but we should take into account the possibility of the confounding.

In addition, all of the subjects were male volunteers. The reason for the deviation might be that the strategy to solve economic games could differ by gender [9], [10] and that its neural basis could be different [8]. Future study also including female volunteers is strongly recommended.

### Conclusion

We observed a significant effect of the cooperative level of the group on punishing behavior for non-cooperators, and the effect on neural activation was significant in the right DLPFC and left ACC.

We for the first time captured the neural mechanism of punishment affected by cooperative social contextual stimuli. It was indicated that the cooperative context, as well as aversive emotion for inequity, is the important factor of punishing behavior. In this respect, the right DLPFC, recruited by social contextual stimuli, plays an important role in punishing behavior.
